# Supplementary material for: Mycobacterium tuberculosis Rv2626c‐derived peptide as a therapeutic agent for sepsis
Source: EMBO Mol Med. 2020 Dec 1;12(12):e12497. doi: 10.15252/emmm.202012497 (PMC7721357; doi:10.15252/emmm.202012497)
Supplement: Supplementary file 1 — Appendix [file EMMM-12-e12497-s001.pdf]

Appendix Data

Mycobacterium tuberculosis Rv2626c-derived peptide as a therapeutic agent for sepsis

Sun Young Kim[1,4], Donggyu Kim[2,4], Sojin Kim[2], Daeun Lee[2], Seok-Jun Mun[1], Eun-I Cho[1], Wooic Son[2], Kiseok Jang[3], Chul-Su Yang[2,*]

[1]Department of Bionano Technology, Hanyang University, Seoul 04673, S. Korea; [2]Department of Molecular and Life Science, Hanyang University, Ansan 15588, S. Korea; [3]Department of Pathology, Hanyang University College of Medicine, Seoul 04673, S. Korea; [4]These authors contributed equally to this work.

Table of content

Appendix Figure S1. Characterization of rRv2626c in macrophages.

Appendix Figure S2. Rv2626c associates with TRAF6 and attenuates Ubiquitination of TRAF6.

Appendix Figure S3. Effects of Tufsin-conjugated rVehicle or rRv2626c-CA on inflammation.

Appendix Figure S4. Therapeutic rRv2626c proteins are uptaken by macrophages in mice.

Appendix Figure S5. Effect of therapeutic rRv2626c proteins on bactericidal and immune cells infiltration in mice.

Appendix Figure S6. Graphical summary of the regulation of TLR signaling pathway mediated by rRv2626c.

Appendix Table S1. List of exact p-values.

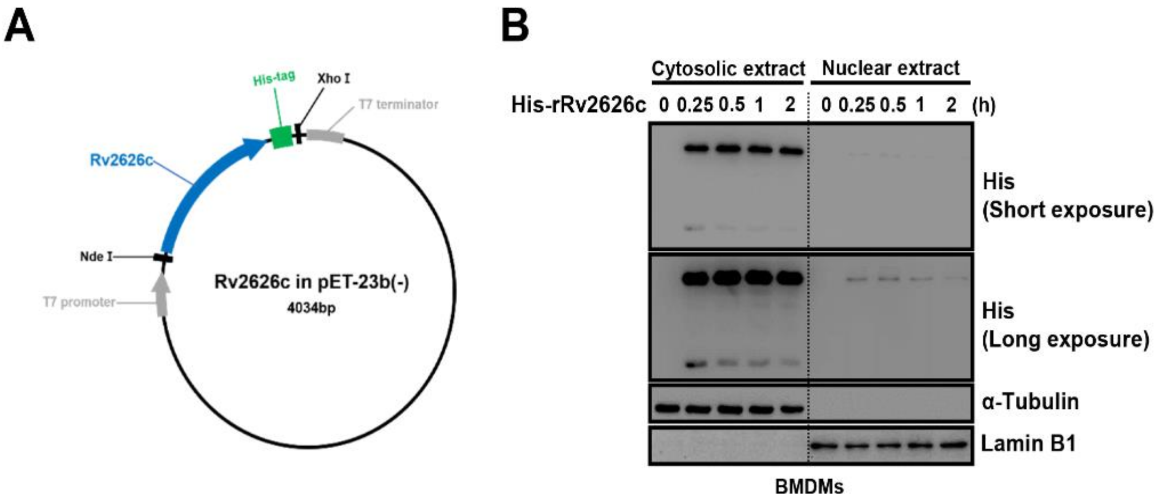

Appendix Figure S1. Characterization of rRv2626c in macrophages.

(**A**) Schematic representation of the cloned Rv2626c in pET-23b(-) vector. (**B**) After BMDMs treated with 2.5 μg/ml rRv2626c for indicated times, cell nuclear and cytoplasmic fractions were separated and analyzed for His-tagged rRv2626c by IB. α-Tubulin was detected as cytoplasmic protein loading controls. Lamin B1 was detected as nuclear loading controls. Data shown are representative of three independent experiments with similar results.

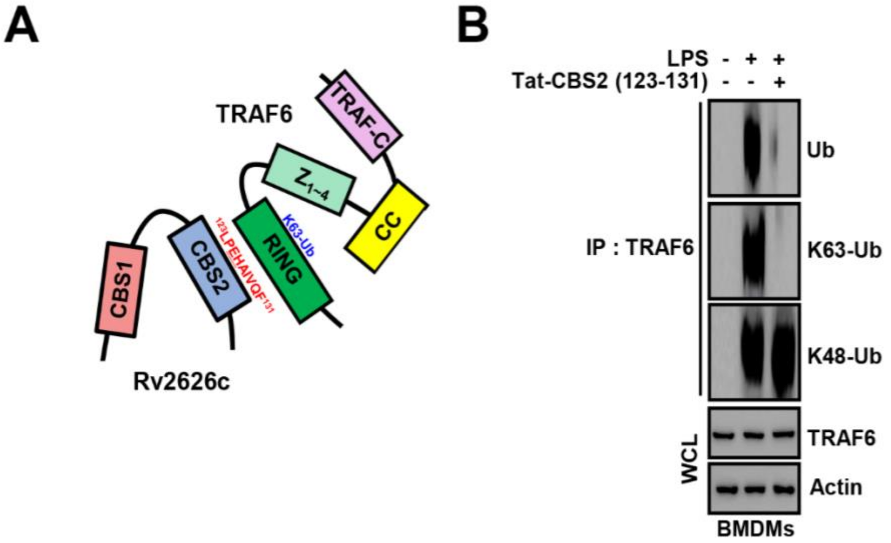

Appendix Figure S2. Rv2626c associates with TRAF6 and attenuates Ubiquitination of TRAF6.

(**A**) Summary of the $_{125}$EH$_{126}$ amino acid with electrically charged side chains of 9 amino acid ($_{123}$LPEHAIVQF$_{131}$) of CBS2 were important for essential and minimal interact with RING domain of TRAF6. (**B**) BMDMs were pretreated with 10 μM Tat-CBS2 peptide for 1 h, and stimulated with 100 ng/ml LPS for 30 min, followed by IP with αTRAF6, IB with ubiquitin, K48-linked ubiquitin, or K63-linked ubiquitin. WCLs were used for IB with αHis, αTRAF6, or αActin. The data are representative of five independent experiments with similar results.

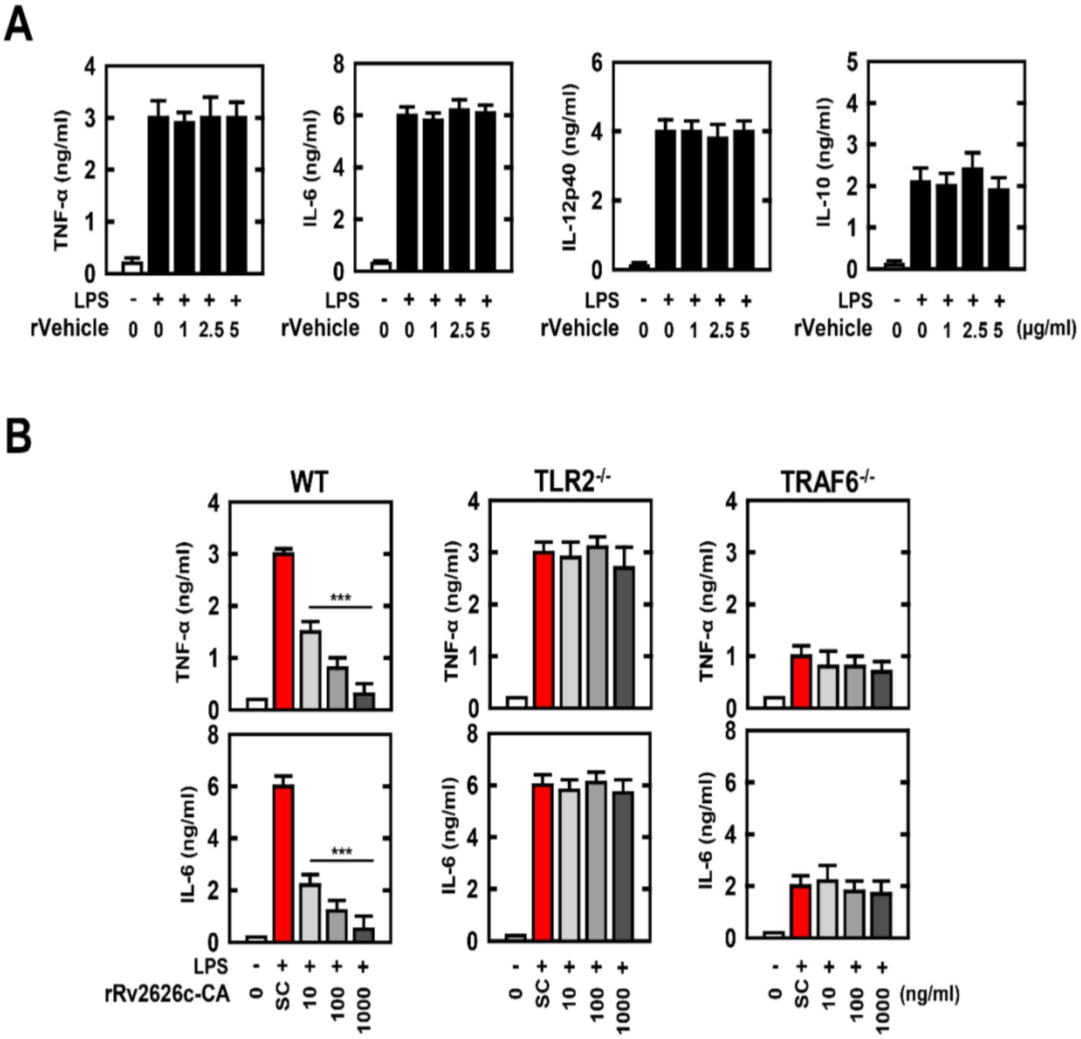

Appendix Figure S3. Effects of Tufsin-conjugated rVehicle or rRv2626c-CA on inflammation.

BMDMs were pretreated with rVehicle (**A**) or rRv2626c-CA (**B**) for 1 h, and stimulated with 100 ng/ml LPS for 18 h. Culture supernatants were harvested, and the levels of TNF-α, IL-6, IL-12p40, and IL-10 were measured by ELISA. Data shown are the means ± SD of three experiments. Statistical significance was determined by Student's *t*-test with Bonferroni adjustment (***P < 0.001) compared with LPS only. SC, solvent control (PBS).

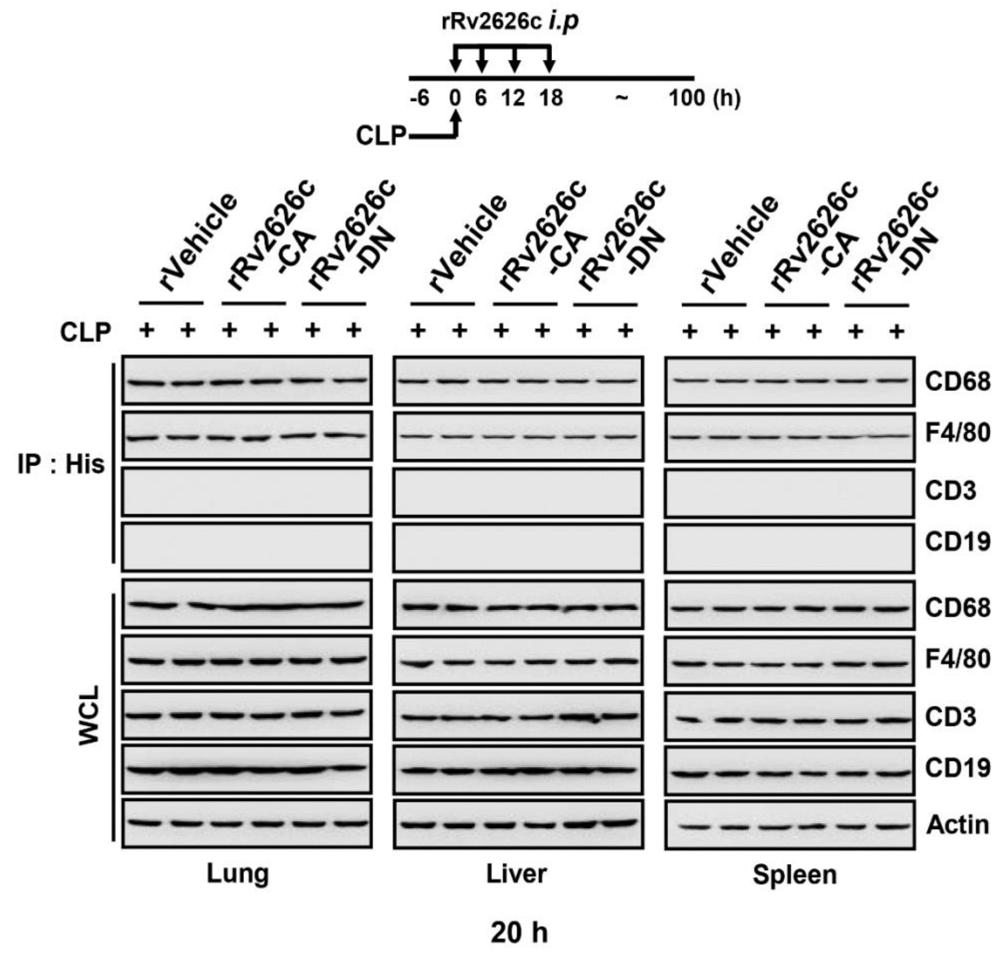

Appendix Figure S4. Therapeutic rRv2626c proteins are uptaken by macrophages in mice.

Schematic of the pharmacokinetic analysis in mice treated with rRv2626c (up). Pharmacokinetic analysis of proteins in the various organs, followed by IP with αHis, and IB with αCD68, αF4/80, αCD3, αCD9, and αActin. The data are representative of three independent experiments with similar results.

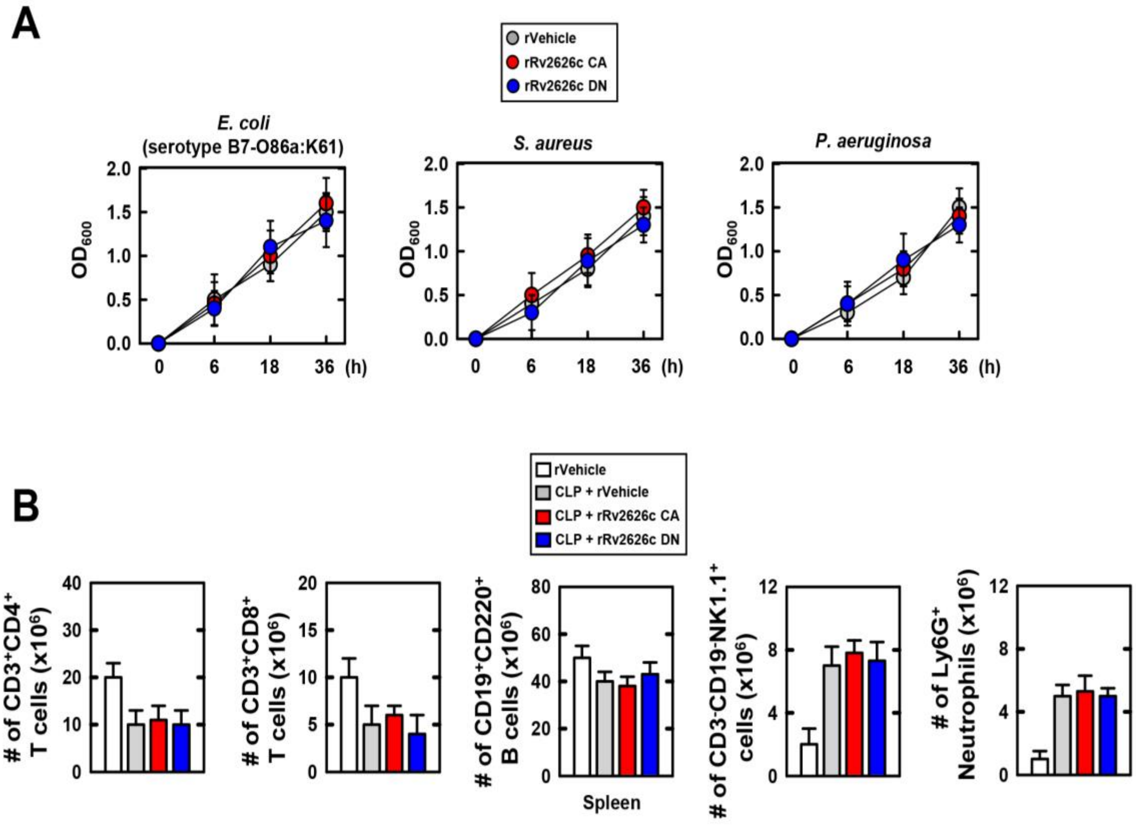

Appendix Figure S5. Effect of therapeutic rRv2626c proteins on bactericidal and immune cells infiltration in mice.

(**A**) Bacteria were cultures in LB broth contained in presence of rRv2626c-CA or its mutants (50 μg/ml) for the indicated times at 37 ºC. Measure the OD_{600} every 6 h. Data shown are the means ± SD of three experiments. (**B**) The percentage of CD3+CD4+ T cells, CD3+CD8+ T cells, CD19+CD220+ B cells, CD3-CD19-NK1.1+ cells, Ly6G+ neutrophils and rRv2626c-CA-His or its mutants were found in the spleen using Fluorescence-activated cell sorting analysis in the background of CLP-induced sepsis. Data shown are the means ± SD of three experiments.

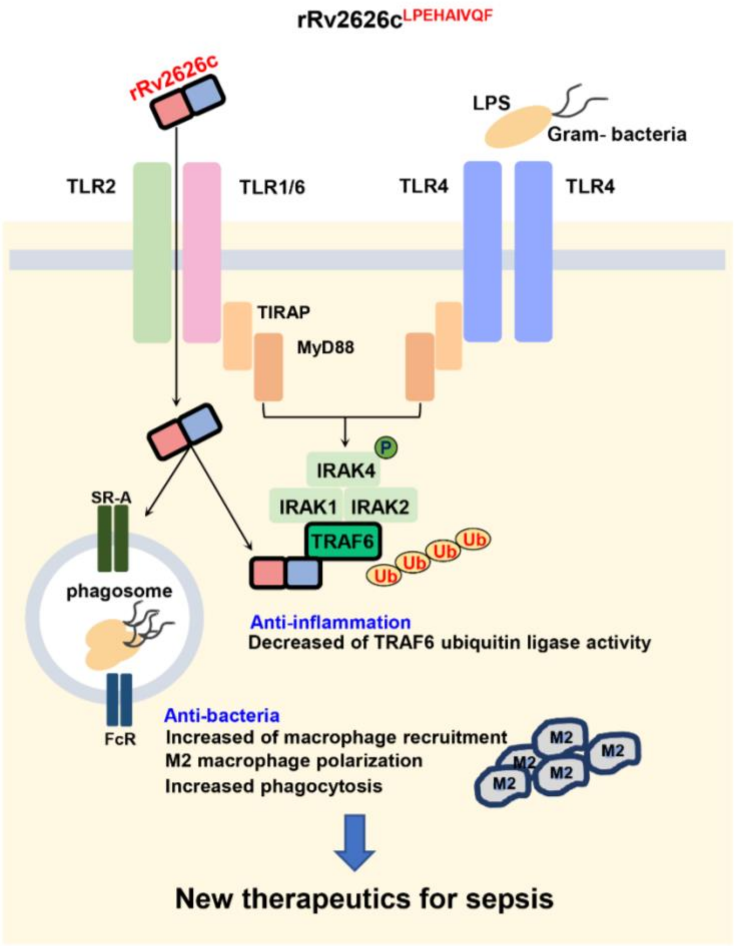

Appendix Figure S6. Graphical summary of the regulation of TLR signaling pathway mediated by rRv2626c.

In macrophages, LPS stimulation triggers activation of cell surface TLR4 and initiates MyD88-dependent signaling pathway. In this pathway, TRAF6 is activated by its K63-linked poly-ubiauitination and promotes downstream signaling cascade that culminates with NF-κB activation and pro-inflammatory cytokine production. CD14-dependent endocytosis of TLR4 induces initiation of endosomal TLR4-TRIF signaling pathway and promotes type I IFN production. In the presence of rRv2626c, rRv2626c enters into the cytosol via TLR2-MyD88 dependent pathway. Cytosolic rRv2626c binds to N-terminal region of TRAF6 through CBS2 domain and inhibits K63-linked polyubiquitination (E3 ubiquitin ligase activity). Furthermore, rRv2626c has anti-bacterial function improved of sepsis. Consequentially, rRv2626c negatively regulates LPS and sepsis-mediated TLR4 signaling pathway.

Appendix Table S1. List of exact p-values.

Figure	Label		p-values
Figure 1E	TNF-α	rVector vs rRv2626c	
		TLR2-/-	0.000531742
		MyD88-/-	0.000274675
		TRAF6/-	0.000183743
		IRAK1-/-	0.000037633
	IL-6	rVector vs rRv2626c	
		TLR2-/-	0.000173633
		MyD88-/-	0.000087736
		TRAF6/-	0.000273763
		IRAK1-/-	0.000478732
	IL-12p40	rVector vs rRv2626c	
		TLR2-/-	0.000778787
		MyD88-/-	0.000658335
		TRAF6/-	0.000636364
		IRAK1-/-	0.000444431
	IL-10	rVector vs rRv2626c	
		TLR2-/-	0.000887733
		MyD88-/-	0.000988874
		TRAF6/-	0.000567872
		IRAK1-/-	0.000046746
Figure 2A	TNF-α	rVector vs rRv2626c	
		6h	0.000765332
		18h	0.000436537
		48h	0.000384764
	IL-6	rVector vs rRv2626c	
		6h	0.000874432
		18h	0.000183735
		48h	0.000087672
	IL-12p40	rVector vs rRv2626c	
		6h	0.000173632
		18h	0.000664743
		48h	0.000988774
	IL-10	rVector vs rRv2626c	
		6h	0.007284732
		18h	0.000736464
		48h	0.000254365
Figure 2B	TNF-α	rVector vs rRv2626c	
		6h	0.016325322
		18h	0.004662255
		48h	0.037565768
	IL-6	rVector vs rRv2626c	
		6h	0.037584843
		18h	0.000345452
		48h	0.008746468

	IL-12p40	rVector vs rRv2626c<br>6h<br>18h<br>48h	0.038546422<br>0.000484758<br>0.000194854
	IL-10	rVector vs rRv2626c<br>6h<br>18h<br>48h	0.000847466<br>0.000565874<br>0.000467842
Figure 2C	p-AKT<br>p-ERK<br>p-p38<br>p-JNK<br>p-IkBα<br>IkBα	rVector vs rRv2626c<br>rVector vs rRv2626c<br>rVector vs rRv2626c<br>rVector vs rRv2626c<br>rVector vs rRv2626c<br>rVector vs rRv2626c<br>rVector vs rRv2626c	0.000173643<br>0.000747332<br>0.000274644<br>0.000284367<br>0.000873248<br>0.000384766<br>0.000452762
Figure 6D	CLP+rVehicle vs CLP+rRv2626c-CA<br>TNF-α<br>IL-6<br>IL-1β<br>IL-12p40<br>IL-10		0.000374632<br>0.000737646<br>0.000263645<br>0.000183734<br>0.000028737<br>0.000183764
Figure 6E	CLP+rVehicle vs CLP+rRv2626c-CA<br>Lung<br>Liver<br>Spleen		0.000264763<br>0.000765322<br>0.000877672
Figure 7A	CLP+rVehicle vs CLP+rRv2626c-CA<br>CFU of blood<br>CFU of peritoneal fluid		0.000026211<br>0.000116365
Figure 7B	CLP+rVehicle vs CLP+rRv2626c-CA<br>Spleen<br>Lung		0.000215124<br>0.000436372
Figure 7C	CLP+rVehicle vs CLP+rRv2626c-CA<br>CD86<br>iNOS<br>CD163<br>Arg1		0.007373633<br>0.000124354<br>0.000484722<br>0.000464532
Appendix Figure S3	WT<br>TNF-α	LPS+SC vs rRv2626c-CA<br>10<br>100<br>1000	0.000837376<br>0.000374622<br>0.000134442
	WT<br>IL-6	LPS+SC vs rRv2626c-CA<br>10<br>100<br>1000	0.000373633<br>0.000272634<br>0.000183732
